# Has universal health insurance reduced socioeconomic inequalities in urban and rural health service use in Thailand?

**DOI:** 10.1016/j.healthplace.2010.06.010

**Published:** 2010-09

**Authors:** Vasoontara Yiengprugsawan, Gordon A. Carmichael, Lynette L-Y Lim, Sam-ang Seubsman, Adrian C. Sleigh

**Affiliations:** a The Australian National University, ANU College of Medicine, Biology & Environment, Building 62, National Centre for Epidemiology and Population Health, Canberra ACT 0200, Australia; b Thai Health-Risk Transition: A National Cohort Study, Sukhothai Thammathirat Open University, Nonthaburi 11120, Thailand

**Keywords:** Universal health insurance, Urban–rural inequality, Health service use, Thailand

## Abstract

This study analyses urban and rural health service use before and after the introduction of the Universal Coverage Scheme (UCS). Using data from the Thai national health surveys of 2001 and 2005, the study utilises age–sex adjusted concentration indices to measure within-area differences in use of health services among populations distinguished by socioeconomic status. Between 2001 and 2005, the UCS substantially reduced Thailand’s uninsured population (from 42.5% to 7.0% in urban areas and from 24.9% to 2.7% in rural areas). The implementation of the UCS changed patterns of health services use, particularly for rural people and the urban poor, by placing greater emphasis on primary healthcare. Relevant policy recommendations should focus on continued improvement of primary health services, and ensuring adequate and timely referrals to secondary and tertiary health services when the need arises.

## 1 Introduction

Population health and economic development have improved significantly in many developing countries over the past three decades. However, development has often been unequal. Consequently, many studies have noted urban–rural inequalities in health behaviours and status (Harpham, 2009; Haynes and Gale, 2000; Paykel et al., 2003; Sabbah et al., 2003) and in health services use (Larson and Hill, 2005; Liu et al., 2007). Using data from the Thai national health surveys of 2001 and 2005, we analyse urban and rural health service utilisation for recent illness and non-maternity conditions requiring hospital admission before and after the introduction of the Universal Coverage Scheme (UCS), and provide evidence to assess the impact of this health insurance initiative on socioeconomic inequalities in health service use in urban and rural areas.

Thailand is in the midst of rapid health and economic transitions. Except during the economic crisis years of 1997–1998, rapid economic growth has over the past six decades brought about a sustained reduction in absolute poverty. Relative poverty (inequality in income distribution) has not, however, followed the same path (Krongkaew, 1993; Warr, 2004). Indeed many regional income disparities that exist in the country have been widening, especially those between urban and rural areas (Sarntisart, 2004). As part of an epidemiological transition, Thailand has moved on from having high levels of maternal and infant mortality and poverty-related disease to a new set of emerging health problems that include chronic diseases and injuries. But although the infant mortality rate has dropped by half in the past 20 years it has fallen more slowly in rural areas, so that the rural–urban ratio has actually widened from 1.3 times in 1964 to 1.5 times in 1985 and 1.8 times in 2005 (NSO, 2006).

The health care system in Thailand consists of both public and private sectors. Under the supervision of the Ministry of Public Health, the public sector has played an important role in health service delivery to the majority of Thai people. Public health facilities include regional hospitals (>500 beds), provincial and other general hospitals (120–500 beds), community hospitals (10–120 beds), and tens of thousands of health centres at sub-district (Tambon) level. Rural residents generally receive health services, including immunization, health promotion services and preventive care, from health centres and community hospitals. For urban residents there are also some health centres and community hospitals, but regional, provincial and other general hospitals mean secondary and tertiary medical services generally are close at hand. Private hospitals and clinics are also mainly located in Bangkok and other cities.

The Thailand Health Profile 2001–2004 and other reports looking back over decades reveal many facets of inequity in the Thai health system (Seubsman et al., 2007; Wibulpolprasert, 2005). In spite of efforts to expand health insurance coverage through various schemes commencing in the 1970s, at the end of the millennium one-third of the Thai population still had no health insurance (Pramualratana and Wibulpolprasert, 2002). Before 2001 there were two public health insurance schemes: a free care for the poor scheme known as the Medical Welfare Scheme (MWS), initiated in 1975, and a subsidised Voluntary Health Card Scheme (VHCS) that was introduced in 1983. Employment-related schemes were the Civil Servant Medical Benefit Scheme (CSMBS), established in 1978 for current and retired civil servants and their dependents (spouse, up to three dependent children and parents), and the Social Security Scheme (SSS), launched in 1990 to cover illnesses and injuries affecting employees in the formal private sector (but not their dependents). Despite the existence of these schemes, Thai studies have documented substantial inequality in health service access, with the poor tending to use health services less when ill and incurring higher expenses relative to their incomes when doing so (Pannarunothai and Mills, 1997). Thailand adopted a Universal Coverage Scheme (UCS) in 2001–2002, covering all its otherwise uninsured citizens (Tangcharoensathien and Jongudomsuk, 2004). The UCS employs a capitation model, with initially either a fee exemption or a minimal co-payment of 30 Baht (∼0.75 USD) per ambulatory visit or hospital admission (Prakongsai et al., 2002). Then, from 2006, the government abolished the co-payment altogether.

Since the implementation of the UCS, several Thai studies have reported inequalities in health status (Yiengprugsawan et al., 2009a, 2007) and in overall use of health services (Coronini-Cronberg et al., 2007; Suraratdecha et al., 2005; Vasavid et al., 2004). Other dimensions of inequalities have also been addressed including healthcare financing, catastrophic payments, as well as impoverishment due to medical care costs. For example, a collaborative effort with the European Union, the EQUITAP (Equity in Asia-Pacific Health Systems) project assessed equity in health financing and delivery in 15 Asia-Pacific countries (van Doorslaer et al., 2007). Benefit incidence analysis has indicated that public subsidies benefited the poor more than the rich when compared to the situation before the UCS (Prakongsai et al., 2006). Another study confirmed that UCS policy not only prevented households from incurring liability for catastrophic health payments, but also protected them from becoming impoverished (Somkotra and Lagrada, 2008) and no system of informal under-the-table payments has emerged (Damrongplasit and Melnick, 2009).

UCS policy requires scheme members to be registered at a primary healthcare facility, and except in an emergency to first access the healthcare system where registered. The primary care network – Contracted Units for Primary Care (CUPs) – acts as gatekeeper to higher level hospitals. This generally means at a health centre in rural areas, but sometimes a community hospital in urban locations if no local health centre exists. UCS patients are then referred up the healthcare system if their medical need warrants it. Bypassing one’s level of registration to go directly to a higher level in non-emergency situations incurs liability for the full cost of treatment at that level out-of-pocket. The primary healthcare infrastructure on which the UCS rests was set in place over 2-3 decades prior to its introduction. In 1979 there were 4088 health centres nationwide at a ratio of 1:10,064 non-municipal (rural) population. By 1987 there were 6992 at a ratio of 1:4964, and by 1997 9477 at a ratio of 1:4173. By 2006 health centres had only increased further to 9762. Community hospital beds numbered just 2540 in 1977, but had risen to 10,800 in 1987, 22,830 in 1997, and 29,780 in 2001 (Wibulpolprasert, 2008). Accordingly, community hospital bed to population ratios also improved in both urban and rural areas; for example from 1:337 to 1:206 in Bangkok and from 1:1511 to 1:759 in the Northeastern region between 1979 and 2002. It is also noted that these Bangkok-Northeast comparisons showed reduced disparities between the richest part of the country (Bangkok) and the poorest region (the Northeast) over that period before the UCS began.

The present study has three objectives: *first*, to describe the patterns of urban and rural use of health services before and after the UCS; *second*, to quantify and assess the magnitudes of inequalities in health service use before and after the UCS; *third*, to compare the patterns and inequalities in health service use between urban and rural areas before and after the UCS.

## 2 Materials and methods

### 2.1 Participants

Data analysed are from the 2001 and 2005 waves of the national Thai Health and Welfare Survey (HWS). Children aged less than 15 years were excluded, leaving for analysis 168,141 adults in 2001 and 52,011 adults in 2005. The 2001 sample was larger because the National Statistical Office wished to produce reports at provincial level for that year (National Statistical Office, 2001).

### 2.2 Measurements

The health outcomes studied are health services use in response to recent illnesses and non-maternity conditions resulting in hospitalisation. English translations of relevant survey questions are as follows: “Have you been ill or not feeling well during the past (2 weeks in 2001; 1 month in 2005)?”, and “Have you been admitted to a hospital during the past 12 months?” These two questions are independent of each other. Admissions for maternity purposes were omitted from the inpatient analysis as pregnancy was not viewed as an ‘illness’, and these admissions were generally reflective of the natural conclusion of a voluntarily initiated biological process, not of a compromised health status the respondent would ideally have avoided. Where an affirmative response was received to either question above, a respondent was asked “What types of health service did you use?” Multiple services were not coded; only the service at which treatment was ultimately obtained, perhaps after referral from a lower level health facility.

It should be noted that because of the order in which questions were asked, the analysis presented is restricted to those reporting illnesses, and no adjustment for ‘need’, or illness, is required. Data on health services use were not obtained from all respondents; only from those who reported being recently ill or hospitalised during the past year. Data were weighted to represent the national age–sex and geographic structures of the Thai population. Stata 9.0 software was used for analysis.

### 2.3 Measures of socioeconomic status

Adult-equivalent monthly income per capita was used to measure socioeconomic status in this analysis, with each child aged less than 15 years weighted as 0.5 of an adult. Total household income was estimated by summing monthly income and monthly income in-kind (i.e., remittances from family members, household agricultural produce) for all household members. We accounted for economies of scale in households with more than one member by raising the adult-equivalent household size to the power of 0.75 (Limwattananon et al., 2007).

### 2.4 Measure of socioeconomic inequality

In recent years, terminological confusion has persisted over the difference between health inequity and health inequality. Inequality and equality can be perceived as dimensional concepts, simply referring to measurable quantities. Inequity and equity, on the other hand, are political concepts, expressing a moral commitment to social justice (Kawachi et al., 2002).

In this study, the concentration index (*C*) was used to measure socioeconomic health inequalities (Wagstaff et al., 1991). When the health outcome is concentrated towards the lower (or higher) end of the socioeconomic scale the concentration index becomes negative (or positive). The larger its absolute value (maximum=1.0), the more pronounced the inequality is. The age–sex structures of samples are known to be confounders in studies of socioeconomic health inequalities. Thus the concentration index were age–sex adjusted (C*), using a procedure as described elsewhere (Kakwani et al., 1997). For binary health outcomes (e.g., use or non-use of a health service), the feasible bounds of the concentration index narrow as the prevalence rate rises. In order to compare observed concentration indices when outcome prevalences differ, the concentration index can be normalised by dividing by 1 minus the prevalence (Wagstaff, 2005). This approach is also adopted here, and normalised concentration indices and are presented as percentages of limiting values for each concentration index.

## 3 Results

### 3.1 Nature of illnesses reported

Around 30% of those reporting recent illness in both 2001 and 2005 reported respiratory ailments, with close to 20% reporting musculoskeletal conditions and about 10% each digestive and cardiovascular conditions. Among those reporting illnesses resulting in hospitalisation, around 21% at both dates reported conditions of the digestive system, roughly 13% cardiovascular conditions, about 10% respiratory illnesses, and 6–8% each of urinary, endocrine/metabolic and musculoskeletal conditions.

### 3.2 Urban and rural descriptive statistics

Despite substantial demographic similarities, rural samples were marginally older in both surveys (Table 1). Proportionately, slightly more rural respondents were aged 45–59 and 60 or older, and fewer were 30–44. Proportions aged 15–29 were virtually identical. Urban samples had slightly more females and rural samples slightly more males. Single persons were substantially more common in urban than in rural areas, and consequently urban respondents were more likely to live in households of 1–2 persons and less likely to live in households with three or more, and especially five or more, family members.

Respondents in rural areas were socioeconomically much worse off than those in urban areas in both surveys. The median adult-equivalent monthly income per capita in urban areas was well over double what it was in rural areas. Mean adult-equivalent monthly incomes per capita also reflected this urban–rural income disparity, and it follows that urban and rural socioeconomic inequalities in this paper are assessed relative to different scales of socioeconomic status. Rural respondents were less well educated as well, being much more likely than urban respondents to have no more than primary education and much less likely to have secondary or higher education.

Distinct differences are also evident in occupational distributions. Urban respondents were much more likely to be professionals and managers, and also more often service workers. Workers in the agricultural and fishery sectors, on the other hand, were overwhelmingly resident in rural areas. Proportions not in the workforce were higher in urban areas, where this group was likely to comprise mainly students and housewives not engaged in family enterprises. Geographically, Bangkok and the remainder of the Central region accounted for 64% of urban respondents at both survey dates. The Northeast contributed 40–41% of rural respondents, followed by the North and the Central region outside Bangkok, with 22–23% each at both survey dates.

### 3.3 Distribution of health insurance

Substantial declines in proportions of respondents with no health insurance coverage occurred between 2001 and 2005 in both urban and rural areas (Table 2). Because rural respondents were poorer, they were much more likely to be eligible for the MWS in 2001 (35.7% compared to 11.5% in urban areas), and this substantially explains the lower rural proportion with no health insurance at that date. After the introduction of the UCS, 23.6% of rural respondents in 2005 were covered by the UCS with fee exempt, compared to only 8.9% of urban respondents.

In 2001, the VHCS covered 26.2% of rural and 9.2% of urban respondents. The difference arose mainly because the VHCS developed out of rural community health projects. In 2005, the largest proportions of respondents in both rural and urban areas were covered by the UCS with co-payment (55.3% and 40.5%, respectively). Because of the nature of their employment, urban respondents were two to three times more likely to be eligible for the CSMBS for public sector workers and the SSS for formal private sector workers in 2001, and this urban–rural differential remained in place under the UCS. 2001–2005 increases in proportions covered by the SSS resulted from a 2002 amendment to the Social Security Act that extended the scheme’s coverage from businesses with five or more workers to those with one or more.

While data in Table 2 do not directly tabulate transfers between health insurance categories over time, they suggest that those previously covered by the MWS mainly transferred to the UCS fee exemption, and the hitherto uninsured and persons covered by the VHCS moved largely to the UCS with co-payment. Some rural MWS members seem also to have moved to the UCS with co-payment, since the rural proportion fee-exempt in 2005 was well below that covered by the free MWS scheme in 2001.

### 3.4 Inequalities in use of health services for recent illnesses

Reported relative frequencies of recent illness increased from 2001 to 2005, most noticeably in rural areas; however, although the reference period doubled between surveys from 2 weeks to 1 month, increases observed in 2005 were much less than two-fold (Table 3). At both dates rural residents were more likely to have been recently ill, the differential more pronounced in 2005 under the UCS.

Buying one’s own medication from a pharmacy was the major source of treatment for recent illness in urban areas, reported by 29.8% in 2001 and 28.9% in 2005. It was also the major source of treatment in rural areas in 2001 (22.3%), but had slipped marginally behind health centres and community hospitals by 2005 (19.8%). Pharmacy use was disproportionately concentrated among the better off in both urban and rural areas at both dates, but the measured socioeconomic inequalities were all small (C*=0.017 in urban areas and C*=0.045 in rural areas in 2001; C*=0.037 in urban areas and C*=0.028 in rural areas in 2005).

Rural respondents reported depending on health centres four to five times more frequently than urban respondents in both 2001 and 2005. However, in both years disproportionately more frequent use of health centres by the poor was more marked in urban areas, as reflected in urban age–sex adjusted concentration indices that were roughly twice as extreme as rural ones (C*=−0.192 in urban areas, C*=−0.090 in rural areas in 2001). Introduction of the UCS saw the magnitudes of these inequalities rise appreciably, by 56.7% in urban areas and 40.7% in rural areas between 2001 and 2005, as health centre clienteles became more sharply focused on the poor (C*=−0.301 in urban areas, C*=−0.123 in rural areas in 2005).

In both 2001 and 2005, the use of community hospitals for recent illness in rural areas was double that reported in urban areas and over that period both use frequencies increased by around 30%. Inequalities associating the poor with community hospital use were particularly substantial for urban areas – some 3–4 times their magnitude for rural areas – but barely changed after introduction of the UCS (C*=−0.319 in urban areas, C*=−0080 in rural areas in 2001; C*=−0.297 in urban areas, C*=−0.065 in rural areas in 2005).

Regional, provincial, or general hospitals were used for recent illnesses by 26.8% of urban respondents and 20.1% of rural respondents in 2001, but by only 19.8% and 9.5%, respectively, in 2005. These drops in patronage, and especially the large decline in rural areas, reflect the UCS directing members initially to primary healthcare facilities, and to regional, provincial, and general hospitals (i.e. ‘higher-level hospitals’) only when medical indications warrant (C*=−0.016 in urban areas, C*=0.056 in rural areas in 2001; C*=0.039 in urban areas, C*=0.125 in rural areas in 2005).

Private clinics, and especially private hospitals, were at both dates used for recent illness more by urban residents, although the urban–rural differential in clinic use in 2005 was marginal. Use of private facilities had increased for both urban and rural residents in 2005 compared to 2001. Both categories of facilities were used disproportionately by the better off in urban and rural areas alike, but this tendency was modest for private clinics (C*=0.070 in urban areas, C*=0.083 in rural areas in 2001; C*=0.054 in urban areas, C*=0.124 in rural areas in 2005) and by contrast marked for private hospitals (C*=0.306 in urban areas, C*=0.364 in rural areas in 2001; C*=0.210 in urban areas, C*=0.474 in rural areas in 2005), in keeping with the affordability disparity between an outpatient visit to a private doctor and admission to a private hospital.

### 3.5 Inequalities in use of health services for illnesses resulting in hospitalisation

Table 4 shows urban and rural hospital admission patterns for non-maternity purposes and associated inequalities in 2001 and 2005. Overall there was a fairly stable hospital use rate involving admissions over the previous 12 months for 5–6% of the population, with the rural admission rate being 16% and 25% higher in the 2 years. As with recent illness, the rural populace had poorer health status. After the introduction of the UCS the patterns of inpatient health service use changed. For both urban and rural areas the proportion of hospital admissions that were to community hospitals rose by over 50%, the compensating declines accruing entirely to regional, provincial, and general hospitals in urban areas, and largely to such hospitals in rural areas. Private hospitals slightly increased their share of urban admissions, but lost share in rural areas. The trend towards community hospitals and away from higher-level hospitals reflects the UCS seeking to channel its members to health service levels appropriate to the seriousness of their medical conditions.

The trend towards greater patronage of community hospitals for inpatient treatment under the UCS was accompanied by a weakening of pro-poor patronage in both urban and rural areas but a continued pro-poor disparity when urban and rural areas are compared (C*=−0.334 in urban areas, C*=−0.126 in rural areas in 2001; C*=−0.231 in urban areas, C*=−0.059 in rural areas in 2005).

For hospitalisation, regional, provincial and general hospital patients were not strongly socioeconomically selected prior to the UCS, and that remained the situation in 2005 (C*=−0.058 in urban areas, C*=0.015 in rural areas in 2001; C*=−0.041 in urban areas, C*=−0.025 in rural areas in 2005). Private hospital patients, on the other hand, were strongly selected for being better off, this tendency weakening under the UCS for urban areas but intensifying sharply for rural areas (C*=0.248 in urban areas, C*=0.229 in rural areas in 2001; C*=0.206 in urban areas, C*=0.360 in rural areas in 2005).

## 4 Discussion

This paper examines patterns of health service use for recent illness and non-maternity conditions requiring hospital admission in rural and urban areas of Thailand in 2001 and 2005, before and after introduction of the UCS. It also examines socioeconomic inequalities in the use of particular types of services within urban and rural areas, and how these changed under the UCS. Between 2001 and 2005, the UCS substantially reduced the proportion of adults without health insurance (from 42.5% to 7.0% in urban areas and from 24.9% to 2.7% in rural areas). Except in emergencies, UCS policy required members to first access primary healthcare facilities at which they were registered—chiefly health centres, but sometimes community hospitals in urban locations. Consequently, the proportionate use of regional, provincial, and general hospitals for both recent illness and non-maternity inpatient conditions substantially fell, especially in rural areas. Clearly the reduction in patronage of these hospitals was an intended product of the diversion of UCS-covered people (mostly poor or of middle socioeconomic rank) to primary healthcare gatekeepers under UCS policy. The use of higher-level hospitals for recent illness became a little more concentrated among the better off, but that was not the case for illnesses resulting in hospitalisation, modest negative concentration indices prevailing. Private clinics and hospitals largely served the better off, and in the case of recent illness this pro-rich inequality became twice as pronounced in rural compared to urban areas under the UCS. In part this reflected weakening of the inequality in urban areas, probably due to the 2002 extension of eligibility for the SSS insurance scheme to smaller private sector enterprises.

Major urban–rural differences in types of health services used for recent illness could be explained largely by the rural proximity and availability of health centres and community hospitals, which are designed primarily to serve a rural clientele, and the urban location of secondary and tertiary services. Markedly more pronounced urban (and less pronounced rural) patronage of health centres and community hospitals by the poor is likely to reflect several things: the ready accessibility of alternative services to the better off with non-UCS health insurance in urban areas; remoteness from, and the travel costs incurred in accessing alternative services in rural areas, which doubtless sees some who could afford them settle for more conveniently located primary services; opportunity costs involved in rural people accessing urban health services; much lower rural coverage by employment-related health insurance schemes that offer direct access to secondary or tertiary health services; perhaps the location of urban poor on city peripheries, from where they may tend to patronize rural health facilities. As already noted, the UCS directed members, and thus particularly the poor, first to primary healthcare facilities—health centres and community hospitals. In rural areas even those without UCS cover may at times use these facilities because they are locationally convenient and their ailments are minor.

For hospital admissions for non-maternity purposes in both urban and rural areas, but more strongly in rural areas, the UCS shifted patients from regional, provincial, and general hospitals to community hospitals. Overall, the inequalities in both urban and rural areas in higher-level hospital admissions were quite small. By contrast community hospital admissions were strongly particularly common among poorer population, especially in urban areas, and private hospital admissions decisively favoured the better off, with a sharp rural strengthening of this tendency under the UCS. The latter trend might be linked to a small 2001–2005 decline in the rural proportion hospitalised in private facilities (Table 4), with previously uninsured persons now covered by the UCS who were moderately well off no longer as apt to opt for private care, and that option consequently more strongly restricted to the wealthy.

Differences in use of health services between urban and rural areas are clearly in part a product of different levels of employment-related health insurance cover. CSMBS and SSS cover were much more common in urban areas. The former insurance scheme facilitates direct access to regional, provincial, and general hospitals, and the latter direct access to private health facilities. Private facilities, especially private clinics in rural areas and private hospitals in urban areas, were used more for recent illnesses in 2005 than in 2001. Both trends, and a weakening of the pro-rich concentration index for urban use of private hospitals, probably reflect the expansion of SSS coverage to smaller enterprises and consequent increases in coverage. Private hospital use for non-maternity inpatient care did not change much during 2001–2005, aside from the previously discussed strengthening of the rural inequality favouring the better off.

There are some limitations associated with this study. Household surveys have inherent possible reporting bias; this has to be taken into account in the interpretation of results (Sen, 2002). In addition, the recall period for recent illness changed from 2 weeks to 1 month between the 2001 and 2005 surveys, which may to some extent compromise direct comparison of health service use rates. Other studies have found that the longer the recall period, the higher the prevalence of underreporting (Byass and Hanlon, 1994; Ramakrishnan et al., 1999). This study is based on income as measure of socioeconomic inequality; other possibilities could be to use combined measures including education, occupation, or assets.

The focus of our study was healthcare utilisation. Additional analysis taking into account supply of health services would be useful. However, we are not aware of any reports indicating oversupply of services under the UCS or of incentives within the system that might produce such an outcome. Undersupply of health services also could have distorted our study findings. We investigated this phenomenon in the post-UCS Thai population in a large national cohort study in 2005 (*n*=87,134). We noted that the common reasons for foregone health service (“long waiting time” and “could not get time off work”) were unrelated to income or health insurance status (Yiengprugsawan et al., 2009b).

It is also the case that data used here identify only the highest service level at which a respondent was ultimately treated. Future analyses identifying the quality of services and multiple levels of health services through which an individual might have been referred may provide more insight into inequalities in service use within urban and rural populations.

## 5 Conclusion

In this paper, we have described the patterns of urban and rural use of health services, quantified and assessed the magnitudes of inequalities, and compared between urban and rural areas before and after the UCS. The findings here complement earlier studies in other middle income countries that are moving towards universal health insurance (Gottret et al., 2008; Wibulpolprasert and Thaiprayoon, 2008). From a policy perspective, several issues can be raised from our Thai experiences and these are set out below.

First, the Thai UCS relies on a solid primary healthcare foundation (Hughes and Leethongdee, 2007; Towse et al., 2004; Wibulpolprasert and Pengpaibon, 2003). The necessary infrastructure was largely set in place during the 2–3 decades preceding its implementation, but it is important that this infrastructure, and the quality of services provided using it, are constantly evaluated and improved to ensure the foundation remains firm (Camilleri and O'Callaghan, 1998; Taner and Antony, 2006).

Second, between 2001 and 2005, the changes in health service use patterns and inequality patterns documented here substantially reflect the gatekeeper function allotted to primary healthcare facilities under UCS policy, and suggest considerable success in channeling inappropriate demand away from higher level public facilities. It is vital that the exercise of this gatekeeper function is closely monitored to ensure it is being performed well, and that if it is not, action is taken to facilitate timely referrals to regional, provincial and general hospitals when optimal medical welfare requires this. Unburdening higher level public health facilities from routine primary care provision is sensible, but must not be allowed to deprive the poor of access to more advanced care when needed. If the referral system acquires a bad reputation, the temptation will be to bypass it, undermining the UCS system and potentially exposing the poor anew to ill-health-induced economic catastrophes and impoverishment (Limwattananon et al., 2007; Somkotra and Lagrada, 2008).

Third, while this analysis has shown that health centres are predominantly patronized by the rural poor with patronage intensifying under the UCS, one should also note the findings for the urban poor. Resort to health centres and community hospitals is far less common in urban than rural responses to recent illness, but is particularly common among poorer population, as is urban patronage of community hospitals for non-maternity illnesses leading to hospitalisation. Health centres and community hospitals catering to urban clients need to be conscious of the important role they play for the least well off in the urban setting, and need to be appropriately resourced.

Finally, it should be noted that the rural populations using health centres and community hospitals are undeniably also deserving of well resourced primary healthcare services. The socioeconomic contexts in which urban and rural inequalities are calculated are different. The more modest rural pro-poor inequalities noted for use of health centres and community hospitals exist within a generally poorer population. As the UCS covers nearly 80% of rural adults compared to just 50% of urban adults, an equitable health service outcome for rural populations is even more dependent on the UCS to enable fair access to quality primary health services than is the case for urban populations.

## Conflict of interest

None declared.

## Figures and Tables

**Table 1:** Summary statistics (percentage distributions unless otherwise indicated) by urban and rural residence: HWS 2001 and 2005. *Sources*: Health and Welfare Surveys 2001 and 2005.

	2001		2005	
	Urban	Rural	Urban	Rural

No. of households	23,070	38,904	8070	12,091
No. of respondents (excluding children aged below 15 years)	63,887	104,254	21,015	30,996

***Demographic characteristics***				
Age (yr)				
Mean (SD)	37.7 (16.0)	38.4 (16.7)	38.5 (16.0)	39.0 (16.6)
15–29	36.6	36.3	34.5	34.5
30–44	33.1	30.9	32.8	30.5
45–59	18.9	19.9	20.6	21.4
≥60	11.4	12.9	12.0	13.6
Sex				
Male	48.3	50.2	48.2	50.2
Female	51.7	49.8	51.8	49.8
Marital status				
Single	33.4	25.8	33.4	24.7
Married	57.4	65.2	57.4	66.0
Divorced, separated, or widowed	9.2	9.0	9.2	9.2
Household size				
Mean	4.3 (2.2)	4.5 (1.9)	4.0 (2.0)	4.2 (1.7)
1	4.3	2.2	5.5	3.3
2	15.4	10.4	17.7	12.9
3	18.7	19.6	20.0	21.6
4	23.2	24.8	23.2	24.7
≥5	38.3	43.0	33.6	37.5

***Socioeconomic characteristics***				
Monthly income (Baht)^a^				
Mean adult-equivalent monthly household income per capita (SD)	6059.5 (5044.7)	2485.3 (2742.0)	8706.9 (9239.4)	3660.6 (4167.1)
Median	4778.2	1699.9	6187.2	2489.7
0–2999 Baht/month	30.7	73.5	20.5	58.9
3000–5999 Baht/month	30.0	18.4	28.0	25.6
≥6000 Baht/month	39.2	8.1	51.5	15.5
Education				
No education	4.1	6.2	3.4	5.8
Primary level	42.4	65.5	36.3	59.4
Secondary level	34.5	23.1	36.9	27.8
Higher level	19.0	5.2	23.3	7.0
Economic activity				
Professionals	16.9	6.1	18.2	7.7
Service workers	36.1	18.0	39.3	22.1
Agriculture and fishery	6.4	43.3	4.9	38.1
Elementary occupation	8.4	7.9	8.1	9.4
Not in workforce (students, housewives, unemployed, disabled)	32.2	24.7	29.6	22.7

***Geographic characteristics***				
Region of residence				
Bangkok	39.9	n/a	40.0	n/a
Central (except Bangkok)	24.0	22.9	24.4	23.2
North	11.4	22.1	11.4	22.3
Northeast	16.1	40.6	15.9	40.2
South	8.5	14.3	8.4	14.3

a 1 USD∼34 Baht.

**Table 2:** Percentage distributions of health insurance coverage by urban and rural residence 2001 and 2005. *Sources*: Health and Welfare Surveys 2001 and 2005.

**Type of health insurance**	**2001**		**2005**	
	Urban	Rural	Urban	Rural
	*N*=63,887	*N*=104,254	*N*=21,015	*N*=30,996

No health insurance	42.5	24.9	7.0	2.7
Medical Welfare Scheme (MWS)	11.5	35.7	n/a	n/a
Voluntary Health Card Scheme (VHCS)	9.2	26.2	n/a	n/a
Universal Coverage Scheme (UCS) fee exempt	n/a	n/a	8.9	23.6
Universal Coverage Scheme (UCS) co-payment	n/a	n/a	40.5	55.3
Civil Servant Medical Benefit Scheme (CSMBS)	15.2	6.0	16.8	7.2
Social Security Scheme (SSS)	18.2	6.0	24.2	10.1
Private insurance	3.4	1.2	2.6	1.2

Total	100.0	100.0	100.0	100.0

**Table 3:** Recent illness, health service use, and concentration indices^a^ for HWS 2001 and 2005. *Sources*: Health and Welfare Surveys 2001 and 2005.

	**Frequency (%)**		**Age–sex adjusted concentration indices (**C*)		**Normalised**^b^**concentration indices (%)**	
**2001**	**Urban**	**Rural**	**Urban**	**Rural**	**Urban**	**Rural**

***2001***						
***N***	**63,887**	**104,254**				
**Recent illness (2 weeks)**	**13.4**	**16.0**				
No service used	7.6	9.8	−0.057	−0.054	−6.2	−6.0
Pharmacies	29.8	22.3	0.017	0.045	2.4	5.8
Health centres	4.7	20.0	−0.192	−0.090	−20.1	−11.3
Community hospitals	8.2	16.5	−0.319	−0.080	−34.7	−9.6
Regional/provincial/general hospitals	26.8	20.1	−0.016	0.056	−2.2	7.0
Private clinics	13.4	9.1	0.070	0.083	8.1	9.1
Private hospitals	9.5	2.1	0.306	0.364	33.8	37.2
**Total**	**100.0**	**100.0**				

***2005***						
***N***	**21,015**	**30,996**				
**Recent illness (1 month)**	**14.7**	**21.2**				
No service used	9.0	9.9	−0.083	−0.042	−9.1	−4.7
Pharmacies	28.9	19.8	0.037	0.028	5.2	3.5
Health centres	4.5	22.6	−0.301	−0.123	−31.5	−15.9
Community hospitals	10.7	21.8	−0.297	−0.065	−33.3	−8.3
Regional/provincial/general hospitals	19.8	9.5	0.039	0.125	4.9	13.8
Private clinics	14.7	14.2	0.054	0.124	6.3	14.5
Private hospitals	12.5	2.3	0.210	0.474	24.0	48.5

**Total**	**100.0**	**100.0**				

a The concentration index is negative (or positive) when the health outcome is concentrated towards the lower (or higher) end of the socioeconomic scale. The larger its absolute value (maximum=1.0), the more pronounced the inequality is. Normalised results are presented as percentages of limiting values for each concentration index.

b Possible values range from −100 to +100.

**Table 4:** Illnesses resulting in hospitalisation, health services use, and concentration indices^a^ for HWS 2001 and 2005. *Sources*: Health and Welfare Surveys 2001 and 2005.

	**Frequency (%)**		**Age–sex adjusted concentration indices**		**Normalised**^b^**concentration indices (%)**	
	**Urban**	**Rural**	**Urban**	**Rural**	**Urban**	**Rural**

***2001***						
***N***	**63,887**	**104,254**				
**Illnesses requiring hospitalisation**	**5.0**	**5.8**				
Community hospitals	13.2	29.4	−0.334	−0.126	−38.5	−17.8
Regional/provincial/general hospitals	55.9	58.4	−0.058	0.015	−13.2	3.6
Private hospitals	30.9	12.2	0.248	0.229	35.9	26.1
**Total**	**100.0**	**100.0**				

***2005***						
***N***	**21,015**	**30,996**				
**Illnesses requiring hospitalisation**	**4.8**	**6.0**				
Community hospitals	20.4	45.3	−0.231	−0.059	−29.0	−10.8
Regional/provincial/general hospitals	47.4	44.2	−0.041	−0.025	−7.8	−4.5
Private hospitals	32.2	10.5	0.206	0.360	30.4	40.2

**Total**	**100.0**	**100.0**				

a The concentration index is negative (or positive) when the health outcome is concentrated towards the lower (or higher) end of the socioeconomic scale. The larger its absolute value (maximum=1.0), the more pronounced the inequality is. Normalised results are presented as percentages of limiting values for each concentration index.

b Possible values range from −100 to +100.
